# Multimodal Recognition of Emotions in Music and Facial Expressions

**DOI:** 10.3389/fnhum.2020.00032

**Published:** 2020-02-11

**Authors:** Alice Mado Proverbio, Elisa Camporeale, Alessandra Brusa

**Affiliations:** Department of Psychology, University of Milano-Bicocca, Milan, Italy

**Keywords:** N400, emotions, multimodal processing, music, facial expressions, cross-modal, neuroaesthetics, neuroscience of music

## Abstract

The aim of the study was to investigate the neural processing of congruent vs. incongruent affective audiovisual information (facial expressions and music) by means of ERPs (Event Related Potentials) recordings. Stimuli were 200 infant faces displaying Happiness, Relaxation, Sadness, Distress and 32 piano musical pieces conveying the same emotional states (as specifically assessed). Music and faces were presented simultaneously, and paired so that in half cases they were emotionally congruent or incongruent. Twenty subjects were told to pay attention and respond to infrequent targets (adult neutral faces) while their EEG was recorded from 128 channels. The face-related N170 (160–180 ms) component was the earliest response affected by the emotional content of faces (particularly by distress), while visual P300 (250–450 ms) and auditory N400 (350–550 ms) responses were specifically modulated by the emotional content of both facial expressions and musical pieces. Face/music emotional incongruence elicited a wide N400 negativity indicating the detection of a mismatch in the expressed emotion. A swLORETA inverse solution applied to N400 (difference wave Incong. – Cong.), showed the crucial role of Inferior and Superior Temporal Gyri in the multimodal representation of emotional information extracted from faces and music. Furthermore, the prefrontal cortex (superior and medial, BA 10) was also strongly active, possibly supporting working memory. The data hints at a common system for representing emotional information derived by social cognition and music processing, including uncus and cuneus.

## Introduction

Neuroaesthetics studies have outlined how music stimulation can clearly convey distinct emotional sensations to the listeners (e.g., joy, sadness, fear), particularly on the basis of piece musical structure, tonality, style and perceptual characteristics (e.g., Peretz, 1998, 2001; Vieillard et al., 2008; Brattico et al., 2011; Koelsch, 2011a, 2014; Bogert et al., 2016; Proverbio et al., 2016; Proverbio and De Benedetto, 2018). A meta-analysis of 41 musical studies has demonstrated that typical emotions such as happiness, sadness, anger, threat and tenderness can be easily decoded with above-chance accuracy by any listener (Juslin and Laukka, 2003). Nordströma and Laukka (2019) have recently shown that emotion recognition in music is a rather fast process. In their study above-chance accuracy was observed for musical stimuli lasting ≤100 ms for anger, happiness, neutral, and sadness recognition, and for ≤250 ms stimuli for the recognition of more complex emotions. Harmonic structure and timing of a musical fragment are promptly processed and interpreted by auditory regions of our brain. It is known that fast rate music tends to convey joyful sensations, whereas slow music tends to transmit sad feelings (Khalfa et al., 2005). Again, Vieillard et al. (2008) demonstrated that while happy excerpts, with a fast tempo and in a major mode were rated as arousing and pleasant, sad/melancholic excerpts, with a slow tempo and in minor mode, were judged as low in arousal. Threatening excerpts, expressed in a minor mode with intermediate tempo, were rated as arousing and unpleasant, while peaceful excerpts characterized by a slow tempo and major mode were perceived as little arousing and pleasant (Vieillard et al., 2008). Going even further, Vuilleumier and Trost (2015) were able to provide the neural underpinnings of complex music-induced emotions (such as, for example, wonder, transcendence, or nostalgia). They found that aesthetic emotions elicited by music shared core features with basic emotions that can be mapped onto independent dimensions of valence (positive or negative) and arousal (high or low) observed in other affective contexts. These findings highlighted the multidimensional nature of emotional reactions that is object of the present investigation.

In an interesting fMRI study (Trost et al., 2012) the neural mechanisms subserving the arising of positive (high arousal) and negative (low arousal) music evoked sensations were investigated indicating the role of the left striate, insula, motor and sensory areas in positive emotions, and of right striate, orbitofrontal (OBF) and ventromedial (vmPFC) prefrontal cortex and hippocampus in negative emotions. Particularly relevant seemed to be the nigro-striatal reward mechanism [including ventral striatum, ventral tegmental area (VTA), caudate and accumbens nuclei, as well as OBF and vmPFC] for mediating music-evoked pleasurable sensations (Blood and Zatorre, 2001; Menon and Levitin, 2005; Ishizu and Zeki, 2011; Salimpoor et al., 2011). On the other hand, amygdala and insula would be more involved in the processing of negative emotions (Gosselin, 2005; Gosselin et al., 2007).

Overall, the neuroaesthetics literature provides robust evidence that a certain variety of music-induced emotions can be reliably recognized by human listeners, regardless of education, exposure and familiarity to music style (e.g., Laukka et al., 2013; Sievers and Polansky, 2013; Egermann et al., 2015). This hints at a biological root for the neural mechanism devoted to the comprehension of music-evoked emotions (see Proverbio et al., 2019 for a discussion on this topic). In this vein, musical stimuli have been used in several multimodal studies where they were compared with emotions derived from the visual modality, and particularly with facial affect (e.g., Spreckelmeyer et al., 2006; Jeong et al., 2011; Hanser et al., 2015; Baranowski and Hecht, 2017). For example, Logeswaran and Bhattacharya (2009) examined how music-elicited emotions can influence subsequent vision-elicited emotional processing, by having neutral, happy and sad faces preceded (primed) by short excerpts of musical stimuli (happy and sad). The results showed that prior listening to a happy (or sad) music enhanced the perceived happiness (or sadness) of a face, regardless of specific facial expression. Similarly, Kamiyama et al. (2013) examined the integrative process between emotional facial expressions and musical excerpts by using an affective priming paradigm. Happy or sad musical stimuli were presented after happy or sad facial images. Participants had to judge the affective congruency of the presented face-music pairs, and incongruent musical targets elicited a larger N400 component than congruent pairs. In this study only two affective expressions (positive vs. negative) were used, so that the decision was dichotomous, thus having a 50/50 probability to be correct. In our paradigm, overt attention was diverted to the detection of neutral adult faces, in order to tap at inner mechanism of extraction and representation of complex emotions in music by using the N400 paradigm, and four different emotional hues.

N400 is a precious tool for investigating semantic and conceptual representations even in unaware subjects. It reflects the semantic analysis of visual (or auditory information) after about 400 ms from the presentation of the stimulus. The N400 peak could represent the process of accessing to semantic information in long-term memory, and/or the integration of this information with previous knowledge. Therefore, N400 amplitude would be greater in anomalous (incongruent) semantic contexts since integration requires more time and cognitive resources with respect to congruent scenarios (Lau et al., 2008). N400 has been effectively used to investigate the semantic processing of language (Kutas and Federmeier, 2011), pictures (Barrett and Rugg, 1990), pictures and words (Nigam et al., 1992), sounds and musical gestures (Proverbio et al., 2015a), skilled motor actions (e.g., basketball, Proverbio et al., 2012), sign language (Proverbio et al., 2015b), words and music (Daltrozzo and Schön, 2009; Goerlich et al., 2011), space and music (*e.g.*, low tones associated with *basement*, or ascending pitch steps associated with *staircase* (Koelsch et al., 2004; Zhou et al., 2014). Overall, the literature suggests the existence of an amodal and shared conceptual system, indexed by N400 response (to incongruity) in different domains, such as language, music, pictures, actions.

To address this issue, in this study EEG/ERPs were recorded in a multimodal audiovisual task featuring visual perception of facial expressions and listening to emotional music. We hypothesized that emotionally incongruent pairs of facial expressions and musical pieces would elicit N400 like responses to emotional mismatch, provided that music was able to clearly convey its emotional meaning in a “abstract” form, even if music was task irrelevant and unattended by viewers.

We also expected that perceptual N170 to faces was modulated by their emotional content, and particularly by distress (Batty and Taylor, 2003; Proverbio et al., 2006; Sun et al., 2017). In addition, we hypothesized that later P300 response was found greater in amplitude to the more arousing (e.g., distress) than less arousing (e.g., relaxation) emotion, since this ERP response has been proven to reflect the degree of emotion-induced arousal (Carretié and Iglesias, 1995; Polich, 2007). As for the auditory stimulation, the ERP literature predicted an effect of musical content on fronto/central N400 response (Koelsch, 2011b). We hypothesized that N400 was greater in response to negative than positive music, and that possibly showed a right hemispheric asymmetry for the processing of negative music, and vice versa, as predicted by neuroimaging studies (e.g., Schmidt and Trainor, 2001).

Visual N170 and P300, auditory N400 and multimodal N400 responses were therefore quantified and analyzed in all individual subjects.

## Materials and Methods

### Stimuli Validation

Twenty non-musician University fellows participated in stimulus validation. They were 8 males and 12 females with a mean age of 29.2 years. Stimuli to be evaluated were visual (facial expressions) and auditory (music tracks), and were presented simultaneously, in different combinations. The visual stimuli consisted of faces of children showing emotional facial expressions of different types; the musical stimuli instead consisted of instrumental tracks transmitting different emotional sensations.

Facial expression were paired with musical excerpts so that they were emotionally congruent or incongruent (half of the times). The emotional value and effectiveness of the combinations was specifically assessed through validation.

#### Visual Stimuli (Faces)

**FIGURE 1:**
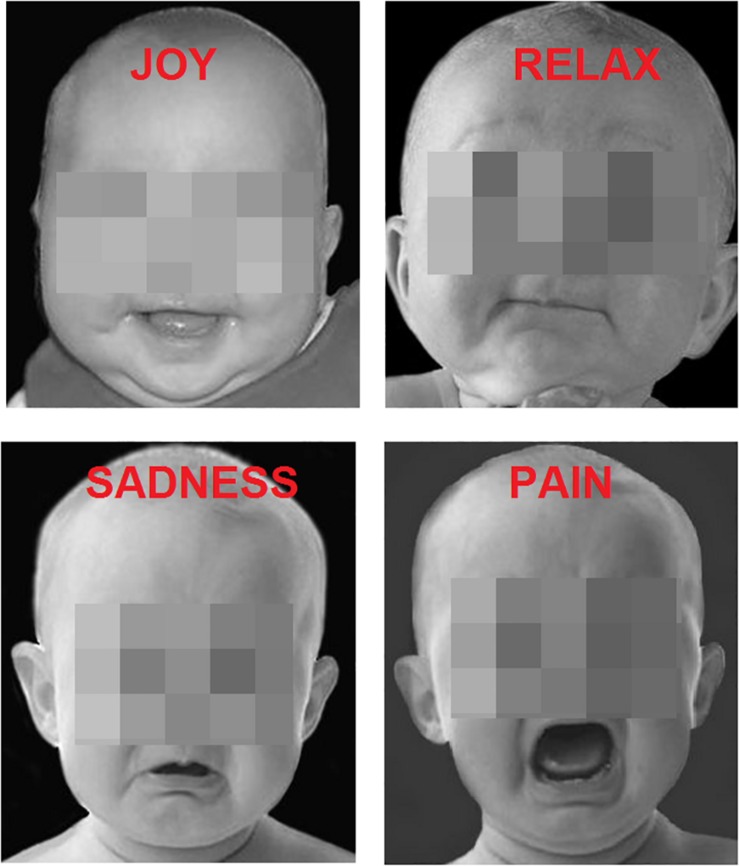
Examples of baby faces displaying the four types of emotions.

The participants were presented with 200 faces of children (Figure 1) (apparent age < 15 months); the images were iso-luminant B/W photographs of infant faces. Each face exhibited a clear-cut, spontaneous, emotional expression caught by the camera, namely: Joy (*N* = 50), Relaxation (*N* = 50), Sadness (*N* = 50), Distress (*N* = 50). The pictures were obtained by a previous study (Proverbio et al., 2007), which validated the universality and comprehensibility of the expressions (see Figure 1 for some examples). The pictures showed only the head of the babies which were all alike. All pictures had a size of 270 × 300 pixels.

### Auditory Stimuli (Music)

Auditory stimulation consisted of 32 music tracks lasting 12 s and validated in the preliminary study by Vieillard et al. (2008). Musical pieces might belong to the following categories, because of their emotional content: Joy (*N* = 8), Relaxation (*N* = 8), Sadness (*N* = 8), Distress (*N* = 8). For any given 12 s musical excerpt 6 different faces were presented.

The music tracks were generated by a computer using the piano tone; they clearly differed for tonality (minor or major) and rhythm (fast or slow) to elicit a different level of arousal in the listener. The happy excerpts were written in a major mode at an average tempo of 137 at metronome (Beat per Minute, BPM), with the melodic line lying in the medium–high pitch range. The sad excerpts were written in a minor mode at an average slow tempo of 46 BPM. The peaceful excerpts were composed in a major mode, had an intermediate tempo (74 BPM). The distress excerpts were composed with minor chords on the third and sixth degree. Although most distress musical pieces were regular and consonant, a few had irregular rhythms and were dissonant. Further information can be found in Vieillard et al. (2008). In our study musical fragments had an average duration of 12.38 s, were normalized with Audacity to −1 Db, and leveled at −70 Db.

The four emotional categories were selected for several reasons: (1) they were clearly comprehensible, (2) were both positive and negative in polarity, (3) were both mild (relaxation and sadness) and strong (distress and joy) in intensity and (4) because a validated set of the same types of facial expressions and emotional music existed.

On the basis of the emotional dimension of the stimuli, 8 types of face-music pairings couplings were created, thus resulting in the emotional congruence or incongruence of the audiovisual stimulation (face/music): joy-distress, relaxation-sadness, sadness-relaxation, distress-joy for the incongruent conditions, and joy-joy, relaxation-relaxation, sadness-sadness, distress-distress for the congruent conditions. Distress baby expressions were paired to “distress” (frightening) musical excepts since stimulus and response are strongly associated (e.g., babies react to fear with tears). Stimulus validation was carried out to ascertain the degree of congruence/incongruity of pairs, and the effectiveness of the face-music couplings. The judges were seated in front of a PC wearing headphones: they were shown Power Point presentation lasting about 25 min, featuring 200 visual stimuli paired to 32 musical fragments, coupled according to their valence and congruence dimensions. To each stimulus pair, the subjects had evaluate their degree of emotional congruence by means of a 4-points Likert scale, where 1 = very incongruent, 2 = incongruent, 3 = congruent, 4 = very congruent.

A repeated measures one-way ANOVA was carried out on the mean scores attributed to the stimuli as a function of the experimental condition (two levels, congruent, incongruent). The ANOVA yielded the significance of condition factor [*F*(1.19) = 262.87, *p* < 0.001], with an average score of 2.03 (incongruent; *SD* = 0.05) for incongruent pairing, and 3.5 (halfway between congruent and very congruent; *SD* = 0.05) for congruent pairings. This preliminary finding confirmed the assumption that both music pieces and facial expressions conveyed a clearly understandable emotional meaning, and that their incongruent mixing was clearly detected by judges.

### EEG Study

#### Participants

Twenty University students (9 males and 11 females) ranging in age from 20 to 26 years participated in the study. Participants were recruited through *Sona System* (a system for recruiting students who earn credit for their Psychology courses by participating in research studies), received academic credits for their participation and provided written informed consent. The data of four participants were excluded because of excessive EEG/EOG artifacts. The final sample comprised sixteen participants (eight males, eight females), aging on average 22.3 years (*SD* = 1.9). All participants had normal or corrected-to-normal vision. They were strictly right-handed as assessed by the Oldfield Inventory and reported no history of drug abuse or neurological or mental disorders. Experiments were conducted with the understanding and written consent of each participant according to the Declaration of Helsinki (BMJ 1991; 302: 1194), with approval from the Ethics Committee of University of Milano-Bicocca (protocol: RM-2019-176).

Data were protected according to EU Regulation 2016/679: article 2/2016 and article 9/2016, concerning the processing of sensitive data and protection of personal data. Participants were informed that their EEG/behavioral data would have been stored in anonymous and aggregate format (combined with those of other participants) for scientific purposes only and for a period not exceeding 5 years.

#### Procedure

The participants were seated inside an anechoic and electrically shielded cubicle about 120 cm away from a PC monitor placed outside the cabin. The images representing children’s emotional faces were presented on a VGA monitor, connected to a compatible IBM-PC computer, located outside the cabin. The onset of auditory stimuli was synchronized with that of visual stimuli at the beginning of each experimental sequence, through an external PC (MacBook Air, Apple) controlling audio clips administration according to the established order of presentation. Participants were instructed to gaze at the center of the screen where a small dot served as a fixation point to avoid any eye or body movement during the recording session. All stimuli were presented in random order at the center of the screen in 4 different, randomly mixed, short runs lasting approximately 1.5 min (plus a training initial run). Stimulus presentation was controlled by *EEvoke* stimulation software (*ANT Software*, Enschede, Netherlands). Each run consisted in the presentation of 50 infant pictures and 8 musical traces. Infant faces were shown for 1000 ms, with an ISI (inter-stimulus Interval) ranging from 600 to 800 ms. Each musical fragment lasted 12 s, and represented a significant musical phrase.

Each participant was provided with written experimental instructions. Before subjecting participants to the actual experimental task, a practical training of sequences was conducted, in which visual and auditory stimuli were used that were not reproduced during the experiment. The training run lasted about 45 s and included the randomly mixed presentation of 24 infants faces (displaying the four types of facial expressions), 4 musical stimuli (one for each of the 4 emotions), and 3 adult faces acting as rare targets.

To keep the subject focused on visual stimulation participants were instructed and trained to respond as accurately and quickly as possible by pressing a response key with the index finger of the left or right hand when they spotted the face of an adult individual. Rare targets were casually intermixed and had a 10% of probability.

#### EEG Recording and Analysis

The EEG was recorded and analyzed using *EEProbe* recording software (*ANT Software*, Enschede, Netherlands). EEG data were continuously recorded from 128 scalp sites according to the 10–5 International System. Sampling rate was 512 Hz. Horizontal and vertical eye movements were additionally recorded, and linked ears served as the reference lead. Vertical eye movements were recorded using two electrodes placed below and above the right eye, while horizontal movements were recorded using electrodes placed at the outer canthi of the eyes, via a bipolar montage. The EEG and electro-oculogram (EOG) were filtered with a half-amplitude band pass of 0.016–100 Hz. Electrode impedance was maintained below 5 KOhm. EEG epochs were synchronized with the onset of video presentation and analyzed using *ANT-EEProbe software*. Computerized artifact rejection was performed prior to averaging to discard epochs in which amplifier blocking, eye movements, blinks or excessive muscle potentials occurred. The artifact rejection criterion was a peak-to-peak amplitude exceeding 50 μV and resulted in a rejection rate of ∼5%. Event-related potentials (ERPs) from 100 ms before to 1000 ms after stimulus onset were averaged off-line. ERP averages were computed as a function of facial expression (regardless of auditory stimulation), for the, therefore so-called, visual N170 and visual P300 responses. Further ERP averages were computed as a function of music emotional content (regardless of visual stimulation) for the, therefore so-called, auditory N400 response. Finally, ERP averages were computed to audiovisual stimuli (regardless of stimuli specific content) as a function of congruence in emotional content (e.g., happy music vs. happy faces = congruent stimulation; distress music vs. happy faces = incongruent stimulation) for the multimodal N400 response.

ERP components were measured when (in time) and where (at which scalp sites on the basis of scalp topography) they reached their maximum amplitudes, and also according to the available literature (e.g., for acoustic potentials central sites were closely monitored). N170 was measured in between 160–180 ms at occipito/temporal sites (P9-P10 and PPO9h-PPO10h), according to previous literature (e.g., Proverbio et al., 2006; Sun et al., 2017). Visual P300 was measured in between 250 and 450 ms at anterior and fronto/central sites (FC1-FC2 and C1-C2). Auditory N400 was measured in between 350-550 ms at fronto-central sites (C1-C2, C3-C4, FCC3h-FCC4h). Multimodal N400 was quantified in between 300–500 ms (N400) at midline prefrontal and inferior frontal sites (Fpz, F5, F6). For each ERP component mean area values underwent distinct repeated-measures ANOVAs whose factors of variability were 3 within-groups factors: Emotion (Joy, Pain, Relaxation, Sadness), Electrode (2 or 3 levels depending on the ERP component of interest), Hemisphere (left, right). Tukey *post hoc* comparisons among means were performed. The effect size for the statistically significant factors was estimated using partial eta squared ($\eta_{\text{p}}^{2}$). The alpha inflation due to multiple comparisons was controlled by means of Greenhouse–Geisser epsilon correction.

Difference Waves (DWs) were also computed by subtracting the ERP waveforms related to congruent stimuli, from those elicited by incongruent stimuli, with the aim of investigating the extraction of the emotional significance of auditory and visual stimuli, regardless of the categories of belonging. The latency of the DW responses considered was between 300 and 500 ms.

#### Behavioral Data

With regard to behavioral data, the percentages of correct responses and response times to targets were analyzed, but not subjected to ANOVA. Accuracy in detecting adult faces was 100% for all of the participants; the average response time was 538 ms (*SD* = 54). This finding suggests that participants were paying close attention to the stimulation provided.

#### SwLORETA Source Reconstruction

Low-resolution electromagnetic tomography (LORETA) was applied to visual N170 and to the difference-waves incongruent-congruent in the N400 time window. LORETA (Pasqual-Marqui et al., 1994) is a discrete linear solution to the inverse EEG problem and corresponds to the 3D distribution of neuronal electrical activity that has a maximal similar orientation and strength (i.e., maximally synchronized) between neighboring neuronal populations represented by adjacent voxels. In this study we used an improved version (Palmero-Soler et al., 2007) of the standardized weighted LORETA. The data were automatically re-referenced to the average reference as part of the LORETA analysis. A realistic boundary element model (BEM) was derived from a T1-weighted 3D MRI data set through segmentation of the brain tissue (Zanow and Knösche, 2004). The source reconstruction solutions were then projected onto the 3D MRI of the Collins brain provided by the Montreal Neurological Institute. The synchronization and coherence tomography incorporates a standard dipole modeling. The probabilities of source activation based on Fisher’s *F*-test were provided for each independent EEG source, the values of which are indicated in the ‘unit’ scale (the greater, the more significant). Both the segmentation and generation of the head model were performed using the ASA software program *Advanced Neuro Technology* (ANT, Enschede, Netherlands). SwLORETA source reconstruction was applied to the ERP waveforms related to the visual N170 (between 160 and 180 ms) as well as to the DWs related to the multimodal N400 (between 300 and 500 ms).

## Results

### Visual N170 (160–180 ms)

**FIGURE 2:**
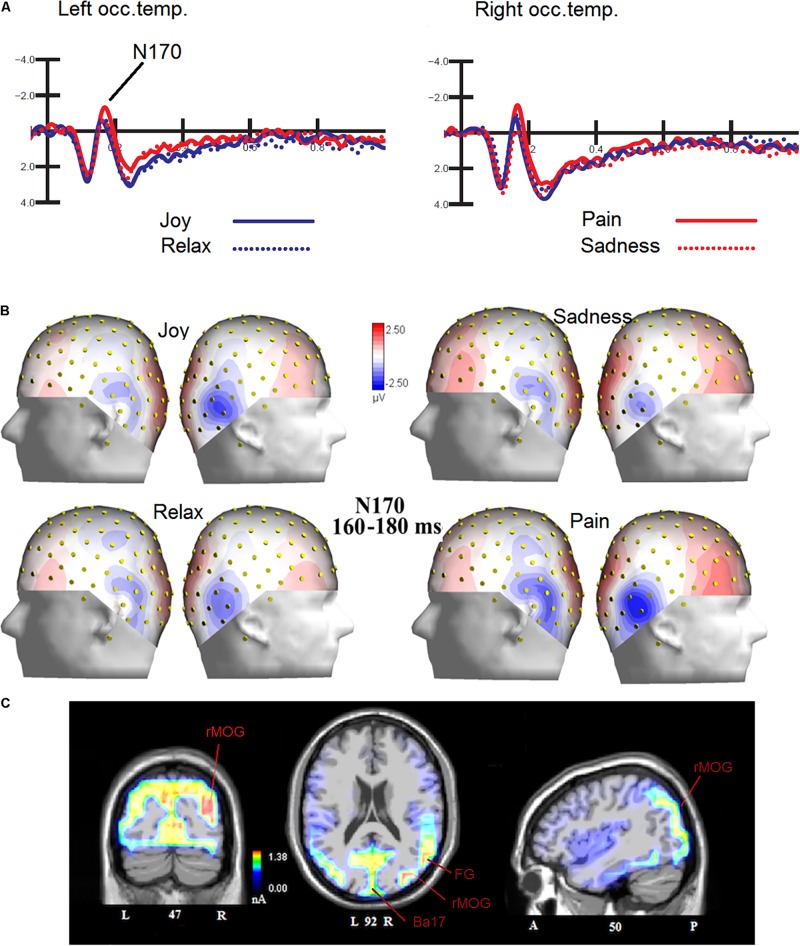
**(A)** Grand-average ERP waveforms elicited by the four types of facial expressions (regardless of music content) as recorded at P9 and P10 sites. **(B)** Isocolor topographic maps of surface N170 voltage measured in between 160-180 ms over the left and right hemispheres, in response to the four types of facial expressions**. (C)** Coronal, Axial and sagittal views of swLORETA activations during face processing at N180 latency range (160–180 ms). The different colors represent differences in the magnitude of the electromagnetic signal (in nA). Numbers refer to the displayed brain slice: L, left hemisphere; R, right hemisphere. A, anterior; P, posterior.

The ANOVA performed on N170 mean amplitude values recorded to visual stimuli (independent of auditory stimulation) showed the significance of emotion [*F*(3,45) = 4.47, *p* < 0.008; ε = 1; $\eta_{\text{p}}^{2}$ = 0.23], with significantly greater N170 responses to distress (−1.68 μV, *SD* = 0.73) than other facial expressions (Joy = −1.0 μV, *SD* = 0.71; Relax = −1.01 μV, *SD* = 0.67; Sadness = −0.68 μV, *SD* = 0.69), as confirmed by *post hoc* comparisons (*p* < 0.005) and as visible in Figures 2A,B. SwLORETA applied to N170 to faces (independently of emotional content) identified as most active areas the right hemispheric occipito/temporal sites (see Table 1). SwLORETA solutions relative to N170 generators are visible in Figure 2C

**TABLE 1:** List of active electro-magnetic dipoles (along with their Talairach coordinates) explaining the surface voltage recorded between 160 and 180 ms post-stimulus (N170 latency range) to faces.

**Magn.**	**T-x [mm]**	**T-y [mm]**	**T-z [mm]**	**Hem.**	**Lobe**	**Gyrus**	**BA**
1.54	40.9	–79.2	12.7	R	O	Middle Occipital	19
1.38	11.3	–88.3	3.0	R	O	Lingual	17
1.31	–28.5	–79.2	12.7	L	O	Middle Occipital	19

*Magn., magnitude of the signal in nA, Hem., hemisphere. L, left; R, right. BA, Brodmann area. O, occipital.*

### Visual P300 (250–450 ms)

**FIGURE 3:**
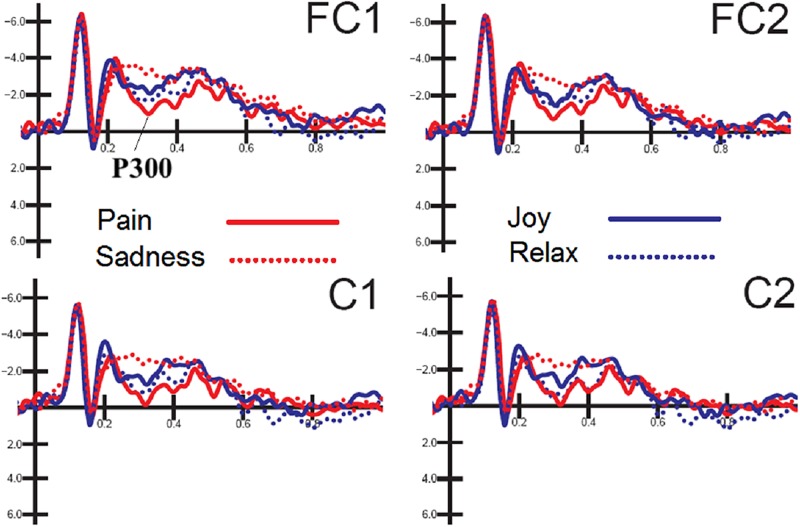
FACES. Grand-average ERP waveforms elicited by the four types of facial expressions (regardless of music content) as recorded at left and right fronto/central sites.

The ANOVA performed on P300 mean amplitude values recorded to visual stimuli (independent of auditory stimulation) showed the significance of electrode factor [*F*(1,15) = 15.57, *p* < 0.001; $\varepsilon =1;\,\eta_{\text{p}}^{2}$ = 0.51] with greater P300 amplitudes over central (C1-C2 = −0.68 μV, *SD* = 0.83) than fronto/central sites (FC1-FC2 = −1.22 μV, *SD* = 0.78), as proved by *post hoc* comparisons (*p* < 0.001). Further significant was the interaction of emotion × electrode × hemisphere [*F*(3,45) = 2.83, *p* < 0.049; = 0.85, adjusted *p*-value = 0.05; $\eta_{\text{p}}^{2}$ = 0.19]. *Post hoc* comparisons showed that P300, especially at right central sites was much greater (*p* < 0.0001) to distress expressions than any other expression (C1 = −0.16 μV, *SD* = 3.24; C2 = −0.08 μV, *SD* = 3.38; FC1 = −0.67 μV, *SD* = 3.11; FC2 = −0.52 μV, *SD* = 3.42), and smaller (*p* < 0.0001) to sad expression than any other expression (C1 = −1.62 μV, DS = 4.25; C2 = −1.4 μV, *SD* = 4.56; FC1 = −2.2 μV, DS = 3.93; FC2 = −1.8 μV, *SD* = 4.12). These P300 modulation as a function of facial expression *per se* can be appreciated by looking at waveforms of Figure 3.

### Auditory N400 (350–550 ms)

**FIGURE 4:**
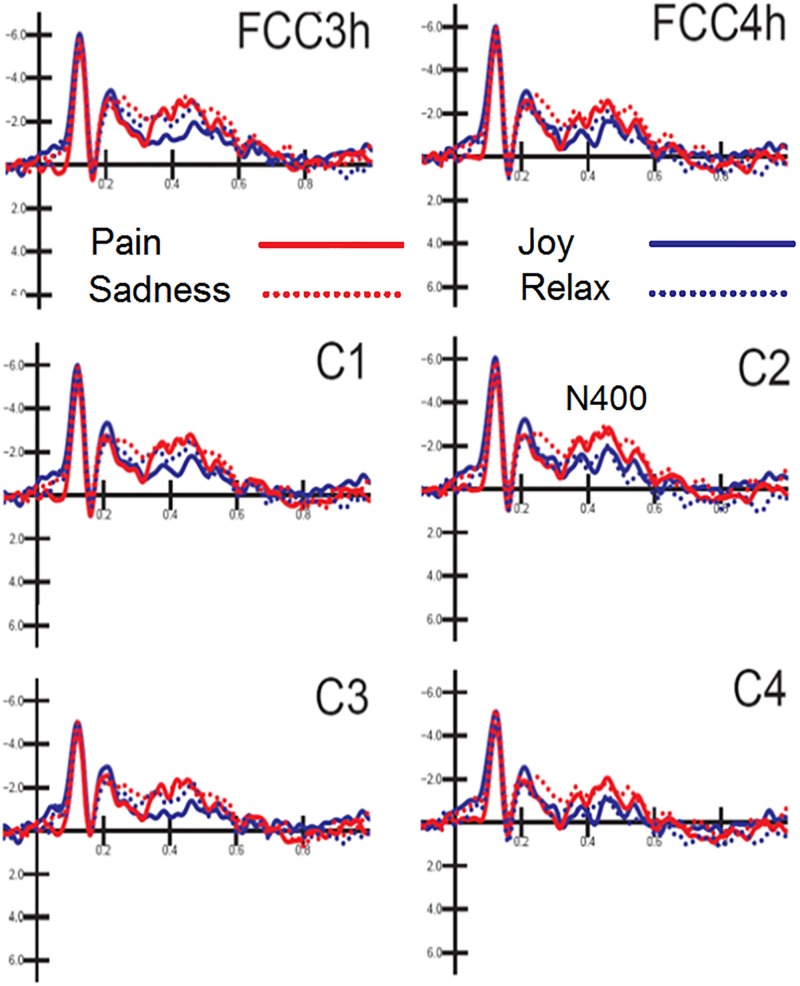
MUSIC. Grand-average ERP waveforms elicited by the four types of emotional content in musical pieces (regardless of facial expressions) as recorded at left and right fronto/central and central sites.

N400 response was measured on ERP waveforms averaged as a function of auditory emotional content (regardless of facial expressions). The ANOVA yielded the significance of hemisphere [*F*(1,15) = 8.73, *p* < 0.009; ε = 1; $\eta_{\text{p}}^{2}$ = 0.36] with greater N400 amplitudes over the left (−1.57 μV, *SD* = 0.48) than right hemisphere (−1.23 μV, *SD* = 0.49). Also significant the interaction between emotion and hemisphere [*F*(3,45) = 4.51, *p* < 0.007; ε = 0.92, adjusted *p*-value = 0.0095; $\eta_{\text{p}}^{2}$ = 0.23]. *Post hoc* comparisons showed that N400 was greater to negative [distress (*p* < 0.001) and sadness (*p* < 0.001)] than positive emotional musical fragments, especially over the right hemisphere (Joy: LH = −0.92 μV, *SD* = 0.56; RH = −0.78 μV, *SD* = 0.6; Relax: LH = −1.54 μV, *SD* = 0.5; RH = −0.89 μV, *SD* = 0.53; Distress: LH = −1.87 μV, *SD* = 0.49; RH = −1.56 μV, *SD* = 0.5; Sadness: LH = −1.95 μV, *SD* = 0.67; RH = −1.72 μV, *SD* = 0.65), as can be appreciated by looking at waveforms of Figure 4.

Also significant the electrode factor [*F*(2,30) = 18.31, *p* < 0.001; ε = 1; $\eta_{\text{p}}^{2}$ = 0.55], showing greater N400 amplitudes over fronto/central (*p* < 0.001) than central sites, as proved by *post hoc* comparisons (C1-C2 = −1.54 μV, *SD* = 0.54; C3-C4 = −1.05 μV, *SD* = 0.44; FCC3h-FCC4h = −1.62 μV, *SD* = 0.47).

### Audiovisual N400 (300–500)

**FIGURE 5:**
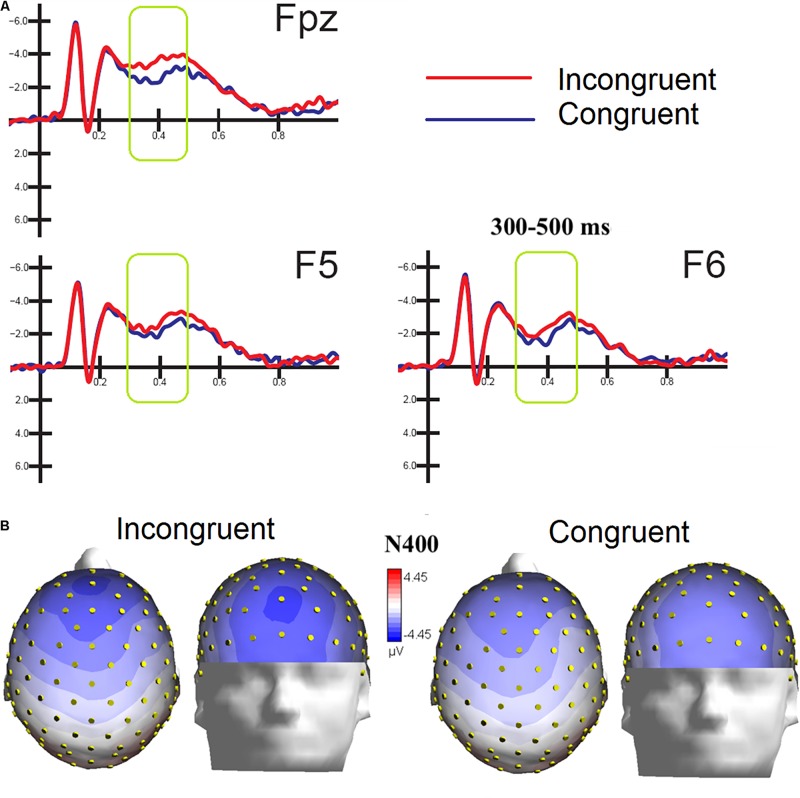
**(A)** MULTIMODAL. Grand-average ERP waveforms elicited by congruent and incongruent audiovisual stimuli (regardless of specific emotional content of music and faces) as recorded at left and right fronto/central an central sites. **(B)** Top and front view of Isocolor topographical maps of surface voltage recorded in between 300 and 500 ms in response to emotionally congruent and incongruent audiovisual stimulation.

ERPs were also averaged as a function of congruence of emotional content of the audiovisual information, regardless of specific emotion conveyed by music and faces. The ANOVA performed on the amplitude values of anterior N400 showed the strong significance of congruence factor [*F*(1,15) = 5.53, *p* < 0.03; ε = 1; $\eta_{\text{p}}^{2}$ = 0.27], with larger amplitudes to incongruent (−2.48 μV, *SD* = 0.44) than congruent stimulation (−1.70 μV, *DS* = 0.44) as clearly visible in Figure 5A. Also significant the factor electrode [*F*(2,30) = 4.18, *p* < 0.025; = 0.96,*adjusted*p*value* = 0.027;$\eta_{\text{p}}^{2}$ = 0.22], showing greater amplitudes (*p* < 0.02) at midline prefrontal than inferior frontal areas (Fpz = −2.56 μV, *SD* = 0.40; F5 = −1.97 μV, *SD* = 0.46 μV; F6 = −1.74, *SD* = 0.45 μV), as was proven by *post hoc* comparisons and visible in topographical maps of Figure 5B.

**FIGURE 6:**
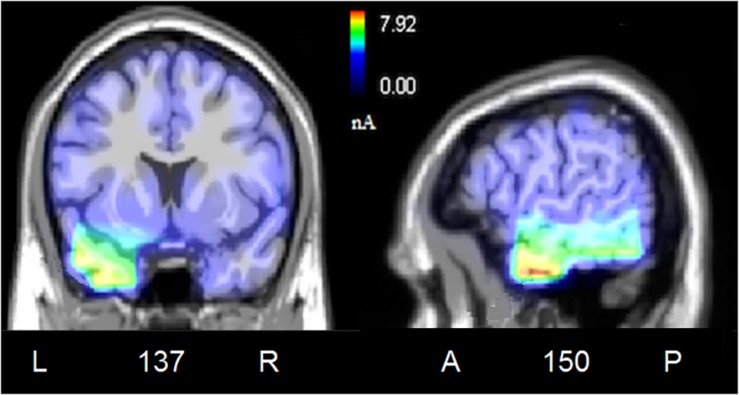
Coronal, and sagittal views of active sources during the processing of incongruent audiovisual information (300–500 ms). The different colors represent differences in the magnitude of the electromagnetic signal (in nAm). The numbers refer to the displayed brain slice in sagittal view: A, anterior; P, posterior. The images highlight the strong activation of inferior and superior temporal cortex in combining the emotional meanings of music and facial expressions.

To locate the possible neural sources of the N400 response, a swLORETA source reconstruction was performed on the difference waves obtained by subtracting the ERPs elicited by the congruent from those elicited by the incongruent condition in the 300–500 ms time window. Table 2 shows the electromagnetic dipoles that significantly explained the surface difference voltages, while the inverse solution is displayed in Figure 6. Overall, the localization of intracranial sources highlighted the contribution of areas extracting and comparing facial and musical affect, particularly the left inferior temporal gyrus (BA20) and the left and right superior temporal gyri (BA38); also active were regions involved in the processing of emotional music (such as the cuneus bilaterally and the left inferior parietal lobule), and the medial prefrontal cortex (BA10).

**TABLE 2:** List of active electro-magnetic dipoles (along with their Talairach coordinates) explaining the surface difference-voltage (incongruent – congruent) recorded between 300 and 500 ms post-stimulus (N400 latency range) to audiovisual stimuli.

**Magn.**	**T-x [mm]**	**T-y [mm]**	**T-z [mm]**	**Hem.**	**Lobe**	**Gyrus**	**BA**	**Presumed function**
7.92	−58.5	−8.7	−21.5	L	T	Inferior temporal	20	Regions extracting and comparing facial and musical affect (e.g., Aubé et al., 2015; Pehrs et al., 2014) Harmonic processing (Peretz, 1998, 2001)
7.39	−38.5	9.1	−27.5	L	T	Superior temporal	38	
4.29	60.6	5.3	2.7	R	T	Superior temporal	22	
7.13	−48.5	−33.7	−23.6	L	T	Fusiform	20	
5.87	11.3	−98.5	2.1	R	O	Cuneus		Processing of frightening music (Koelsch et al., 2018)
5.58	−8.5	57.3	−9.0	L	F	Superior frontal	10	Working memory, Music processing (Bogert et al., 2016)
5.49	1.5	65.3	7.9	R	F	Medial frontal	10	
4.41	−48.5	−61.8	41.2	L	P	Inferior parietal lobule	39	Processing of frightening music (Koelsch et al., 2018)
4.32	21.2	−8.0	−28.9	R	Limbic	Uncus	36	Emotion

*Magn., magnitude of the signal in nA; Hem., hemisphere; L, left; R, right. T, temporal; O, occipital; F, frontal; P, parietal.*

## Discussion

The aim of this study was to investigate how the conceptual incongruity between facial expressions and musical pieces that expressed different emotions was implicitly processed by unaware participants. The more general assumption was indeed that music was able to clearly convey emotional meanings (Panksepp and Bernatzky, 2002; Brattico and Pearce, 2013), so that the concurrent presentation of incongruent information might trigger a N400 response to semantic incongruence, as the one recorded in linguistic (Kutas and Federmeier, 2011), motor (Proverbio and Riva, 2009), musical (Koelsch, 2011b) or perceptual (Barrett and Rugg, 1990) contexts. To this end, pictures depicting emotional facial expressions in infants (Proverbio et al., 2007) and musical fragments belonging to the same four emotional categories as the faces (Vieillard et al., 2008) were created and validated. Stimulus validation demonstrated that music pieces and facial expressions conveyed a clearly understandable emotional meaning, and that their incongruent mixing was clearly detected by subjects.

### Emotional Content of Facial Expressions

The analyses carried out on the visual N170 component showed differences in the processing of the emotions expressed by the faces, specifically N170 was greater in amplitude to distress than other expressions. This result fits with previous literature showing larger N170s to negative than positive or neutral expressions (Batty and Taylor, 2003; Proverbio et al., 2006; Sun et al., 2017). To identify the most active dipoles during the coding of facial expressions, a swLORETA inverse solution (*standardized weighted Low Resolution Electromagnetic Tomography*) was applied to the surface potentials recorded in the time window between 160 and 180 ms: the right middle Occipital Gyrus (MOG, BA 19) was the most active dipole, along with left MOG and primary visual cortex. This findings agrees with previous literature (Haxby et al., 2000; Gobbini and Haxby, 2007; Fusar-Poli et al., 2009) showing the role of the so-called “occipital face area” in the processing of faces, and especially face details. The emphasis on the local element (i.e., a single face element, such as a wrinkle, a skin fold, eye sockets, etc…) might possibly be related to the task, which consisted in analyzing face age (see also Wiese et al., 2012) to detect and respond to adult faces. Althought N170 it is generally larger over the right hemisphere, it is notable that it was more prominent over the right to negative emotions.

The later visual P300 (between 250 and 450 ms), averaged as a function of facial expression, was strongly modulated by emotional content, being it larger to the most arousing emotions (distress), with an intermediate amplitude for joy and relaxed/neutral expressions and smaller for sadness displays. This gradient in neural response possibly reflects an effect of face emotional intensity and induced arousal level (Carretié and Iglesias, 1995; Polich, 2007). This finding is consistent with the previous literature showing larger P300s to emotional than neutral expressions (Lang et al., 1990; Proverbio et al., 2006).

### Hemispheric Asymmetry for Positive vs. Negative Emotions

As for the processing of music content, auditory N400, recorded over the fronto/central area in between 350 and 550 ms, was greater to negative (distress and sadness) than positive emotional musical fragments, especially over the right hemisphere. This right-sided hemispheric asymmetry for processing negative emotions agrees with previous neuroimaging literature showing how the processing of positive and joyful music mostly engage the left frontal cortex, whereas sad or fearful music would mostly engage the right frontal cortex (Schmidt and Trainor, 2001). Again neurological studies in patients with unilateral brain lesions provided evidence of a dominance of the left hemisphere for positive emotions and of the right hemisphere for negative emotions (Ahern and Schwartz, 1979; Reuter-Lorenz and Davidson, 1981; Rodway et al., 2003; Gainotti, 2019). Consistently Davidson (1992) provided evidence that the left prefrontal cortex (PFC) would be more involved in positive and enjoyable stimuli inducing an approach to appetitive stimuli, whereas the right PFC would be more involved in processing aversive and negative stimuli.

### Multimodal Processing of Affective Information

The analyses of grand-average ERP waveforms computed as a function of stimulus congruity, therefore reflecting a multimodal processing, showed a strong N400 effect for the presentation of incongruent pairs. N400 amplitude would be greater in anomalous (incongruent) semantic contexts since integration requires more time and cognitive resources with respect to congruent scenarios (Lau et al., 2008). The present findings are in agreement with previous ERP literature combining musical and facial information and finding larger N400s to emotionally incongruent pairs (Kamiyama et al., 2013), as well as to other types of incongruent multimodal information. For example N400 has been found larger to incongruent pairs of pictures and words (Nigam et al., 1992), sounds and musical gestures (Proverbio et al., 2015a), words and music (Daltrozzo and Schön, 2009; Goerlich et al., 2011), space and music (Koelsch et al., 2004; Zhou et al., 2014). The above studies suggest the existence of an amodal and shared conceptual system, indexed by N400 response to incongruity in different multimodal domains. The present data showed that emotional content of music (appropriately selected) can induce rather distinctive meanings, as clearly comprehensible as facial expressions.

To assess which were the cortical areas responsible for extracting and processing the emotional significance of audiovisual stimuli, difference-waves were computed by subtracting the ERPs elicited by the congruent stimuli from those elicited by incongruent stimuli, and analyzed in the N400 time window (between 300 and 500 ms). The inverse swLORETA solution (*standardized weighted Low Resolution Electromagnetic Tomography*) applied to the N400 response showed as most active dipole the left inferior temporal gyrus (ITG, BA 20), which is anatomically adjacent to the medial temporal gyrus (MTG) identified as the neural generator of linguistic N400 (McCarthy et al., 1995). Very active were also found the bilateral Superior Temporal Gyrus (STG, BA 38/22) and the left Fusiform Gyrus (FG, BA 20), specialized in the coding of the emotional content of music and faces (e.g., Pehrs et al., 2014; Aubé et al., 2015) and harmonic processing (STG, Peretz, 1998, 2001). Indeed, multimodal audiomotor neurons located in the posterior superior temporal sulcus (pSTS) and in the medial temporal gyrus (MTG) respond both to sounds and to visual images of objects and animals (Wright et al., 2003). Again, a specific region located posteriorly and ventrally to STS, named the *temporal visual speech area* (TVSA), seems particularly responsive to auditory and visual speech stimuli as it is also at the basis of audiovisual McGurk illusion (Bernstein and Liebenthal, 2014; Proverbio et al., 2018).

The STG’s role in integrating audiovisual information and in the extraction of affective properties from both sensory modalities has been explored by Aubé et al. (2015) in an fMRI study aimed at investigating the neural correlates of processing specific basic emotions (fear, sadness and joy), expressed through music, vocalizations and facial expressions. They found that the STG was deeply involved in the response to both happy and frightening music, the activation signal being modulated by intensity and arousal. Similarly, in a study in which faces were presented during listening of strongly emotional music (namely, *Pathetic symphony* by Tchaikovsky) it was found that the medial prefrontal cortex (BA10) and the STG were strongly involved the combined processing of facial and musical affective information (Proverbio and De Benedetto, 2018). Again, a recent neuroimaging study (Pehrs et al., 2014) have investigated how the brain integrates the visual information of a movie with its musical soundtrack into a coherent percept. At this purpose dynamic kissing scenes from romantic comedies were presented during fMRI scanning. The kissing scenes were either accompanied by happy music, sad music or no music. The presence of music enhanced activation signals in multisensory integration network consisting of fusiform gyrus, amygdala and anterior superior temporal gyrus (aSTG). Again Baumgartner et al. (2006a, b) explored brain activity during the cross-modal presentation of affective images (sad, fearful) in two conditions, alone or with emotionally congruent musical traces. They found that the main areas involved in the cross-modal (multisensory) integration between emotional images and music tracks were the MTG and the temporal pole. The findings outlined above are generally coherent with the present data, except for the amygdala activation that cannot unfortunately be detected through EEG signals via LORETA.

Other brain areas found to be active in the processing of emotional audiovisual content, in our study, were the right cuneus and the left inferior parietal lobule (IPL, BA 39). Intriguingly Koelsch et al. (2018) found that both regions were active during the processing of fearful music. Also active according to swLORETA were the left superior frontal gyrus and the right medial frontal gyrus, commonly active during music processing (Bogert et al., 2016; Proverbio and De Benedetto, 2018) and reflecting stimulus coding and working memory.

On the basis of the data obtained in this study it is possible to conclude that the extraction and integration of the emotional content of multimodal stimuli takes place automatically (on task-irrelevant information) in a very short time, after about 400 ms from the presentation of the stimuli. This result confirms the extraordinary ability of music to communicate emotions clearly, and distinctively as emotional facial expressions. A discussion about the biological bases of such an innate ability can be found in a recent electrophysiological study comparing the comprehension of spontaneous vocalizations (e.g., laughs and crying) vs. instrumental music (Proverbio et al., 2019). Both stimulation types involved brain areas shaped for processing the human voice and its affective modulations. Indeed it has been suggested that music universality derives from the existence of a common neural mechanism for the comprehension of the emotional content of music, vocalizations (e.g., laughter and crying) and speech prosody (Panksepp and Bernatzky, 2002; Proverbio and Santoni, 2019; Proverbio et al., 2019), mostly relying on fronto/temporal areas. The literature suggests that music and vocalizations use similar patterns of acoustic cues to express emotions (Juslin and Laukka, 2003; Paquette et al., 2013), which might explain some universal and pretty innate brain reaction to music, regardless of cultural factors such as: education, familiarity or aesthetic taste.

The present data show how the brain is highly capable of integrating emotional information coming from different sensory modalities, to form a coherent conceptual representation comparable to semantic meaning of information, and how this mainly involves the MTG and the STG, the superior and medial frontal gyri, uncus, parietal and limbic areas. A right hemispheric dominance for processing negative (distress and sadness) vs. positive emotions (joy and relaxation), was also found at anterior areas, as indexed by N400 response to music.

One study’s limitation might be the somewhat limited sample size, comprising 20 participants in the EEG recording session but only 16 after the EEG artifact rejection procedure. The merits of this study, compared to the previous ones are to have clarified the communicative power of music and facial expressions with a symmetrical and balanced mode of stimulation, with ultra-validated stimuli and with an implicit paradigm to detect the automatic mechanisms of extraction of emotional meaning, without directing or conveying subjective interpretations. The data show a certain universality of some musical parameters in inducing specific emotional sensations.

## Data Availability Statement

The datasets generated for this study are available on request to the corresponding author in case of a scientific cooperation.

## Ethics Statement

The studies involving human participants were reviewed and approved by the Ethical Committee of University of Milano-Bicocca. The patients/participants provided their written informed consent to participate in this study.

## Author Contributions

AP conceived and planned the experiment. EC and AB prepared the stimuli, carried out the EEG recordings, and performed the statistical analyses. AP interpreted the data and took the lead in writing the manuscript. All authors provided critical feedback and helped shape the research, analysis and manuscript.

## Conflict of Interest

The authors declare that the research was conducted in the absence of any commercial or financial relationships that could be construed as a potential conflict of interest.
